# *SICOT-J:* Why another Orthopaedic Open Access journal?

**DOI:** 10.1051/sicotj/2015001

**Published:** 2015-02-13

**Authors:** Jacques Caton, Hatem Galal Said

**Affiliations:** 1 Clinique Orthopédique Emilie de VIALAR Lyon France; 2 Assiut University Hospital Assiut Egypt

Over the last few years, Orthopaedics has been inundated with Open Access journals, the continued quality and survivorship of which is uncertain. SICOT, the only international society of Orthopaedics and Traumatology has therefore decided to participate and take a lead in this rapidly evolving field. *SICOT-J* has now been launched. It is backed by an internationally renowned Editorial Board and we seek quality publications.

*SICOT-J* is an Open Access journal which publishes original clinical, basic and translational research in orthopaedic surgery and traumatology. SICOT-J will accept, subject to peer review, review articles, original articles, research articles, surgical techniques, case reports, congress proceedings, editorials and letters to the Editor.

As *SICOT-J* is published under Open Access conditions, all the articles will also be published quickly by the Open Access Repository (OAR) of publicly accessible scientific articles, in Biomedical and Life Science journals and by PubMed Central which is hosted by the US National Institute of health’s National Library of Medicine (NIH/NLM).

We invite you to submit your articles to *SICOT-J* by visiting the website of the journal at www.sicot-j.org. We hope that many of you will support the Journal as it is with your quality articles that the journal’s impact in the field of orthopaedics and traumatology will quickly flourish.

The Editors-in-chief

Jacques Caton

Hatem Said

